# Testing universality of Feynman-Tan relation in interacting Bose gases using high-order Bragg spectra

**DOI:** 10.1038/s41377-023-01103-8

**Published:** 2023-02-28

**Authors:** Yunfei Wang, Huiying Du, Yuqing Li, Feng Mei, Ying Hu, Liantuan Xiao, Jie Ma, Suotang Jia

**Affiliations:** 1 State Key Laboratory of Quantum Optics and Quantum Optics Devices, Institute of Laser Spectroscopy, Shanxi University, Taiyuan, China; 2 Collaborative Innovation Center of Extreme Optics, Shanxi University, Taiyuan, China; 3 Hefei National Laboratory, Hefei, China

**Keywords:** Atom optics, Micro-optics

## Abstract

The Feynman-Tan relation, obtained by combining the Feynman energy relation with the Tan’s two-body contact, can explain the excitation spectra of strongly interacting ^39^K Bose-Einstein condensate (BEC). Since the shift of excitation resonance in the Feynman-Tan relation is inversely proportional to atomic mass, the test of whether this relation is universal for other atomic systems is significant for describing the effect of interaction in strongly correlated Bose gases. Here we measure the high-momentum excitation spectra of ^133^Cs BEC with widely tunable interactions by using the second- and third-order Bragg spectra. We observe the backbending of frequency shift of excitation resonance with increasing interaction, and even the shift changes its sign under the strong interactions in the high-order Bragg spectra. Our finding shows good agreement with the prediction based on the Feynman-Tan relation. Our results provide significant insights for understanding the profound properties of strongly interacting Bose gases.

## Introduction

Interactions are at the heart of the most intriguing correlated quantum phenomena, which are intractable when treated in full microscopic detail. While a lot of universal relations, which are independent of the details of interactions at the microscopic scale, allow greatly simplify the description of interaction effect in quantum many-body systems. Ultracold atomic gas is a fundamental paradigm for exploring universal physics^1–3^, where the interatomic interaction is characterized by the atomic *s-*wave scattering length and its strength can be tuned via Feshbach resonances^4^. For example, Tan’s universal relation was introduced for connecting the dependence of the energy on the scattering length to the strength of two-particle short-range correlations, where Tan’s contact parameter characterizes the probability of finding two colliding atoms with very small separation^5–11^.

In the elementary excitation of an interacting atomic Bose-Einstein condensate (BEC), the Bogoliubov dispersion relation was given for describing the linear response of excitation energy shift to the strength of the interaction in weakly interacting regimes^12,13^, which was verified in two-photon Bragg spectra^14–17^. However, the breakdown of Bogoliubov theory was observed in the excitation spectra of strongly interacting ^85^Rb BEC^18^. Among the subsequent theoretical interpretations^19–23^, the Feynman-Tan relation was proposed for obtaining a good explanation for the backbending dispersion exhibited in ^39^K BEC with tunable interactions^24^. Owing to the significance of universal relation for the intellectual understanding of interaction-dominated exotic phenomena, it is of particular interest to test whether the Feynman-Tan relation is universal for describing strongly correlated behavior in other atomic systems, since the resonance frequency shift in this relation is inversely proportional to atomic mass. Nevertheless, extending the application of Feynman-Tan relation to different atomic species has so far remained out of reach.

Here our experimental goal is to test the universality of Feynman-Tan relation in strongly interacting Bose gases of ^133^Cs atoms with large mass difference compared to the previous results of ^39^K atoms. We measure the high-momentum excitation spectroscopy of ^133^Cs BEC with widely tunable interactions by using the second- and third-order Bragg spectra, in which the large momentum transfers are involved in the stimulated four- and six-photon processes in comparison to the general two-photon Bragg spectroscopy^14–18,24–30^. In the high-order Bragg spectra, we observe the backbending of the frequency shift of excitation resonance in the moderate interaction regions, and even the shift changes its sign from positive to negative at the strong interactions. Our results show good agreement with the prediction based on the Feynman-Tan relation, and this provides the significant evidence for extending the application of Feynman-Tan relation to different atomic systems.

## Results

Interactions affect the property of atomic gases, and the previous Bogoliubov theory provides the basic framework of modern approaches to BEC with the tunable mean-field interaction^12,13^. The Bogoliubov dispersion relation for the elementary excitations in an interacting BEC is given by

1 $$\varepsilon \left( p \right) = \sqrt {\frac{{p^2}}{{2m}}\left( {2U + \frac{{p^2}}{{2m}}} \right)}$$

where *p* = *ħq* is the momentum transfer in the elementary excitation with the reduced Planck’s constant *ħ* and the wave vector *q*, *m* is the atomic mass, and the mean-field interaction energy is *U* = 4*πħ*^2^*ρa*/*m* with the density *ρ* and the *s*-wave scattering length *a*. For the BEC in a harmonic trap, the averaged atomic density can be obtained by the local density approximation with that the Thomas-Fermi radius of condensate in the *q* direction is larger than the excitation wavelength^16^. In the particle-like excitation with *p*^2^/(2 *m*) ≫ 2*U*, the Bogoliubov approximation gives the interaction-induced frequency shift ∆*ω*_B_ = *ω* − *ω*_0_ = 4*πħρa/m*, where *ω* = *ε*(*p*)*/ħ* and *ω*_0_ = *ħq*^2^*/*(2 *m*) correspond to the actual excitation energy and the free-particle kinetic energy, respectively. In the following experiment with $\sqrt {\rho a^3} < < 1$, the Lee-Huang-Yang correction in the excitation energy of ground BEC can be ignored, and the Bogoliubov approximation is always valid^13^.

When the atomic interactions become sufficiently strong, the beyond mean-field effect observed in ^85^Rb BEC deviates the Bogoliubov dispersion relation^18^. While the recently proposed Feynman-Tan relation can capture the two-photon Bragg spectra of ^39^K BEC^24^, where the Feynman energy relation is used to obtain the excitation energy^31^. In the Bragg scattering process, the static structure factor *S*(*q*), which is obtained by integrating the structure factor *S*(*q, ω*) over *ω*, is the Fourier transform of the density correlation function^16^. For the low density with $\sqrt {\rho a^3} < < 1$, the Feynman energy relation is given as

2 $${\omega (q) = \hbar q^2/\left( {2m} \right)/S\left( q \right)}$$

In the excitations of Bose gases with strong short-range interactions in the deep inelastic regime of large-*q* momentum transfer, *S*(*q*) can be expressed in terms of the universal two-body contact:

3 $${S(q) = 1 + \frac{C}{{8\rho q}}\left( {1 - \frac{4}{{\pi aq}}} \right)}$$

where *C* is Tan’s two-body contact density and reflects the probability for two atoms to be at the same point in space^5–7^. By inserting the contact density of *C* ≈ (4*πρa*)^2^ in Eq. (3), the absolute value of static structure factor is |*S*(*q*)| ≈ 1, and the resulting energy shift is given as ∆*ω* = (1/*S*(*q*) − 1)*ω*_0_^23,24^. Because of 1*/S*(*q*) *−* 1 ≈ 1 *- S*(*q*), the Feynman-Tan relation gives the interaction-induced frequency shift.

4 $${{\Delta}\omega _{{{{\mathrm{FT}}}}} = \frac{{4\pi \hbar \rho a}}{m}\left( {1 - \frac{{\pi qa}}{4}} \right)}$$

For the limit of *qa* → 0, the interaction-induced frequency shift ∆*ω*_FT_ in Eq. (4) is equivalent to the prediction based on the Bogoliubov theory. However, ∆*ω*_FT_ dose not change monotonously with increasing *a*. As indicated in Eq. (4), ∆*ω*_FT_ will decrease after achieving the maximum value and then change its sign under the strong interactions. The backbending phenomenon was observed in the previous experiment of ^85^Rb atoms^18^, where *a* was increased to *a* ~ 2.5/(*πq*). In Ref. ^24^, a homogeneous ^39^K BEC with the low density allows to measure the resonance frequency shift for the larger *a*, and the sign change of ∆ω_FT_ was observed under the strong interactions. Considering the significance of universal relation for understanding strongly interacting Bose gases, it is highly desired to test the universality of Feynman-Tan relation in other atomic systems with the large mass difference relative to ^39^K atoms, because of the dependence of ∆ω_FT_ on *m* in Eq. (4).

**Fig. 1:**
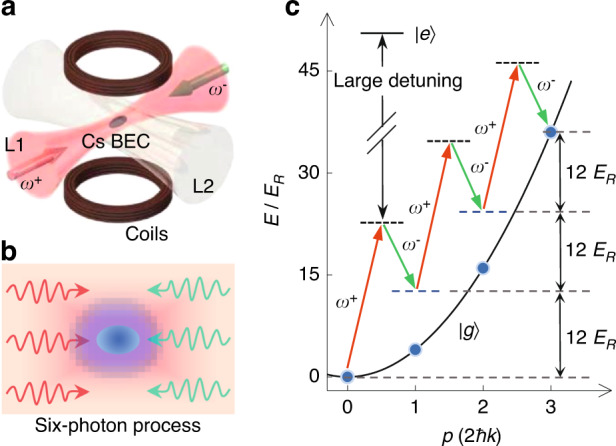
Experiment setup and schematic for high-order Bragg spectra. **a** Sketch of experimental setup. A ^133^Cs BEC is prepared in a quasi-1D optical trap that consists of two nearly orthogonal 1064-nm laser beams (L1 and L2) with the different 1*/e* radius. A pair of counter-propagating Bragg lasers, which are formed by retro-reflecting the trap laser beam (L1), are used to illuminate the optically trapped BEC for implementing Bragg spectra. **b** Schematic of third-order Bragg diffraction involved with the six-photon process. The atom absorbs three photons from the incident laser beam and simultaneously radiates three photons into the retro-reflected laser beam. **c** Illustration of energy diagram in the third-order Bragg resonance. Three stimulated Raman transitions are simultaneously driven for directly coupling the zero-momentum BEC to the momentum state *p* = 6*ħk*, and the energy difference is evenly divided into each transition

Our experiment starts with a ^133^Cs BEC of *N* = 4 × 10^4^ atoms in the hyperfine state |*F* = 3*, m*_*F*_ = 3 > , which features a broad Feshbach resonance to continuously tune atomic *s*-wave scattering length^32–34^. As shown in Fig. 1, the BEC is confined in a quasi-1D optical trap, which is comprised of two nearly orthogonal 1064-nm laser beams (L1 and L2) with the ratio of 1*/e* radius ~1:6 and the wavelength of *λ* = 1064 nm. The laser beam L1 mainly provides the strong radial confinement, and the trap frequencies are (*ω*_*x*_, *ω*_*y*_, *ω*_*z*_) = 2*π* × (125, 96, 10) Hz, where *z* represents the propagation direction of the laser beam L1. The Bragg spectra are implemented by illuminating the BEC with a pair of counter-propagating laser beams, which is formed by retro-reflecting the trap laser beam L1^35–39^. Relative to the fixed frequency *ω*^+^ of incident laser beam, the frequency *ω*^−^ of retro-reflected laser beam is precisely tuned for engineering the frequency detuneing ∆*ω*_*res*_ = *ω*^+^ − *ω*^−^ for the Bragg diffractions.

In Fig. 1b, we show the schematic for a stimulated six-photon process involved in the third-order Bragg diffraction, where the zero-momentum BEC is directly coupled to the high-momentum state *p* = 6*ħk* with the wave vector of Bragg laser *k* = 2*π/λ*. The condensed atom absorb three photons from the incident laser beam and simultaneously radiate three photons into the retro-reflected laser beam, accompanying with the large momentum transfer 6*ħk*. Figure 1c shows the energy diagram of third-order Bragg resonance, where three pairs of counter-propagating photons have the same frequency difference of ∆*ω*_*res*_ = 12*E*_*R*_/*ħ* with the one-photon recoil energy *E*_*R*_ = *ħ*^2^*k*^2^*/*(2*m*). In the second-order Bragg resonance, the condensed atoms are coupled to the momentum state *p* = 4*ħk*, and two pairs of counter-propagating photons have the same frequency difference of ∆*ω*_*res*_ = 8*E*_*R*_/*ħ*.

**Fig. 2:**
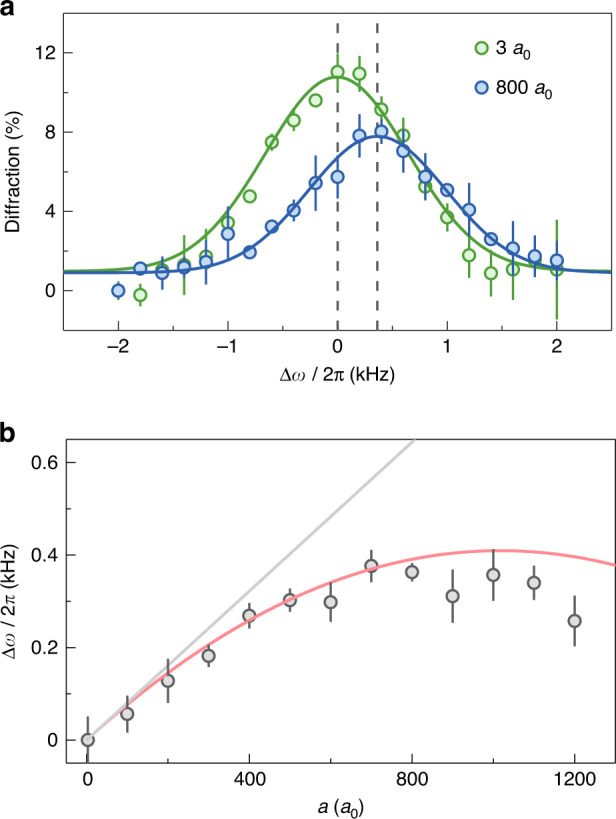
Two-photon Bragg spectra of BEC with tunable interaction. **a** Two-photon Bragg spectra for two different scattering lengths (in unit of Bohr radius of *a*_0_). Fraction of atomic BEC diffracted into the momentum state *p* = 2*ħk* as a function of the frequency shift ∆*ω*, which is referenced to the noninteracting free-particle energy 4*E*_*R*_*/ħ*, after 1 ms Bragg pulse. The resonance shift is determined by a Gaussian fit to the data (solid lines). Error bars denote the standard errors. **b** Dependence of the frequency shift ∆*ω* extracted from the fitting in (**a**) on the scattering length *a*. The grey solid line is obtained by the Bogoliubov theory, and the red solid line is the prediction based on the Feynman-Tan relation in Eq. (4). The averaged density of *ρ* = 1.6 × 10^13^/cm^3^ is taken in the theoretical calculation. Error bars denote the fitting errors

We prepare the BEC at the scattering length of *a* = 210 *a*_0_, where *a*_0_ is the Bohr radius, and then ramp *a* in 6 ms to the target value at which we perform the Bragg diffraction for 1 ms. Figure 2a shows the two-photon Bragg spectra with the momentum transfer *ħq* = 2*ħk* for two different scattering lengths. The diffracted fraction of atoms is plotted as a function of frequency shift, which is normalized by referring the frequency difference in the excitation resonance without interaction ∆*ω* = ∆*ω*_*res*_ − 4*E*_*R*_/*ħ*. The maximal diffracted fraction is kept around 10%. The interaction-induced frequency shift can be determined by the Gaussian fit to the data. We clearly observe that the interaction with *a* = 800 *a*_0_ gives rise to the positive shift relative to the zero shift in the noninteracting limit.

In Fig. 2b we show the dependence of the frequency shift ∆*ω* obtained via two-photon Bragg spectra on the scattering length. For weak interactions with *a* ≤ 400 *a*_0_, the data shows good agreement with the Bogoliubov dispersion relation, where ∆*ω* is linearly dependent on *a*. For the larger *a*, our measurement shows a significant deviation from the Bogoliubov theory, and ∆*ω* bends down under the strong interactions. Instead, the theoretical prediction based on the Feynman-Tan relation in Eq. (4) captures the variation of ∆*ω* with *a*. In the experiment, although the broad Feshbach resonance allows to obtain the maximum scattering length of *a* ~ 1800 *a*_0_^4^, the three-body loss of atoms limits the applied scattering length within *a* ≤ 1200 *a*_0_^40^.

To compromise with the reachable maximum scattering length, the sign change of frequency shift under the strong interactions may be demonstrated by using large-*q* excitation spectra according to Eq. (4). In Refs. ^41,42^, the second- and third-order Bragg diffractions were theoretically proposed for a large momentum transfer in the interferometry of ultracold atoms^43–47^. We use the second- and third-order Bragg spectra with the stimulated four- and six-photon processes (see Fig. 1b, c) to obtain large momentum transfers with 4*ħk* and 6*ħk*, respectively.

**Fig. 3:**
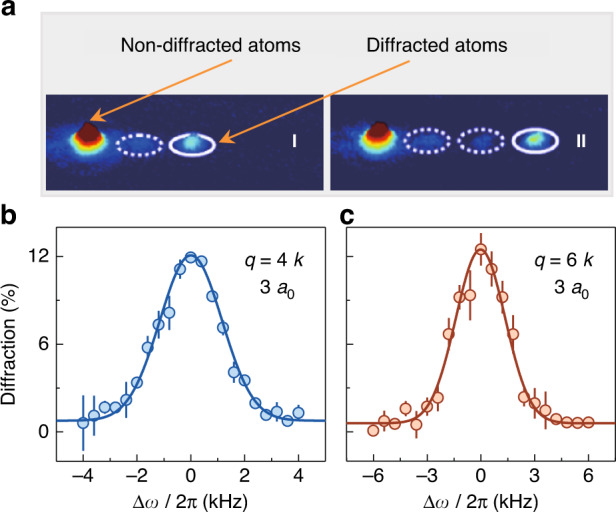
High-order Bragg spectra. **a** Second- and third-order Bragg diffractions correspond to the stimulated four- (I) and six-photon (II) processes with the momentum transfers *ħq* = 4*ħk* and 6*ħk*, respectively. Typical absorption images are taken after the 1 ms Bragg pulse and 22 ms time-of-flight, and about 10% atoms are coupled to the high-momentum states. **b**, **c** Diffracted faction of atoms as a function of frequency shift ∆*ω* normalized to the frequency difference *ħq*^2^*/*(2*m*) in the noninteracting limit. The resonance shift is determined by a Gaussian fit to the data (solid lines). Error bars denote the standard errors. In all panels, the scattering length is fixed at *a* = 3 *a*_0_

Figure 3a shows the absorption images taken after the 1 ms second- and third-order Bragg diffractions and 22 ms time-of-flight, and about 10% atoms with the momenta *p* = 4*ħk* and 6*ħk* are diffracted to the different positions in momentum space. We show the second- and third-order Bragg spectra for *a* = 3*a*_0_ in Fig. 3b, c. The fraction of atoms diffracted to the high momentum is measured as a function of frequency shift, which is normalized to the frequency difference in the noninteracting limit with ∆*ω* = 2(∆*ω*_*res*_ − 8*E*_*R*_/*ħ*) in Fig. 3b and ∆*ω* = 3(∆*ω*_*res*_ − 12*E*_*R*_/*ħ*) in Fig. 3c. The frequency shift of excitation resonance corresponding to the maximum diffraction fraction can be determined by using the Gaussian function to fit the data.

**Fig. 4:**
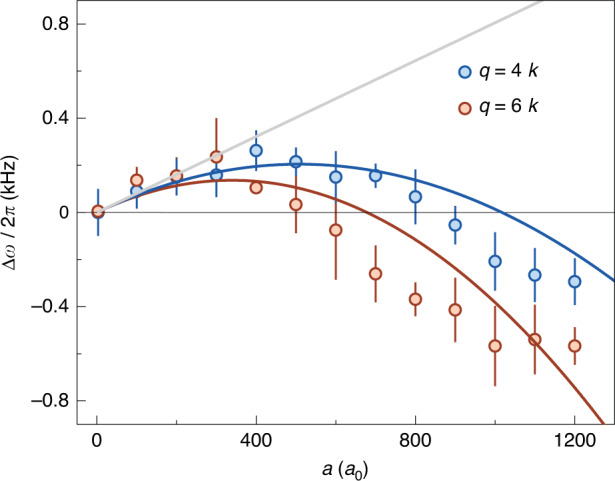
Variation of frequency shift ∆*ω* obtained by using high-order Bragg spectra with the scattering length *a*. The shifts ∆*ω* under different scattering lengths are extracted from the second- and third-order Bragg spectra with the large momentum transfers *ħq* = 4*ħk* and 6*ħk*. The grey solid line is obtained by the Bogoliubov theory, and both the blue and orange solid lines show the prediction based on the Feynman-Tan relation. In comparison to the dramatic deviation from the Bogoliubov theory, the Feynman-Tan relation shows good agreement with the data. The averaged density of *ρ* = 1.6 × 10^13^/cm^3^ is used for the theoretical calculations. Error bars denote the fitting errors

We further measure the second- and third-order Bragg spectra under the different interactions, and obtain the frequency shift of excitation resonance by performing the Gaussian fits as shown in Fig. 3b, c. In Fig. 4, we plot the frequency shift ∆*ω* for two different momentum transfers of 4*ħk* and 6*ħk* as a function of scattering length *a*, and the maximum available value of *qa* is about 2.25 for *q* = 6*k*. In compared to the low-*q* two-photon Bragg spectroscopy, ∆*ω* arrives the maximum value at the relative low *a*, because this critical *a* is given as *a* = 2/(*πq*) (see Eq. (4)). Most importantly, we clearly observe that ∆*ω* changes its sign from positive to negative under strong interactions. The theoretical prediction based on the Feynman-Tan relation reasonably agrees with the data in Fig. 4. Moreover, both the experiment and theory show that the position of crossing point with ∆*ω* = 0 shifts toward the lower *a* as *q* increases, where the zero-crossing position is given as *a* = 4/(*πq*) in Eq. (4). Note that, the discrepancy between the experiment and theory is likely caused by the reduction of atomic density, which results from the thermalization of atomic BEC during the high-momentum excitations under the strong interactions.

In compared to ^39^K atoms in the previous experiment^24^, ^133^Cs atoms have a larger mass and provide a good platform to test the influence of atomic mass on the interaction-induced frequency shift in the Feynman-Tan relation. The quantitative explanation for the dependence of ∆*ω* on *a* in Figs. 2b and 4 verifies the inverse proportional relationship between the atomic mass and resonance frequency shift in Eq. (4). Our results also illustrate that the Feynman-Tan relation can be used for the description of excitation of a harmonically trapped interacting Bose gases with the assumption of local density approximation, although the Feynman-Tan relation is given with the homogenous density. In addition, we compare the data obtained by the high-order Bragg spectra with the Bogoliubov dispersion relation (grey line) in Fig. 4, and the deviation becomes larger with increasing *q* in comparison with the result in Fig. 2b. This indicates that the Bogoliubov theory fails to give the role of the momentum transfer in the interaction-induced frequency shift in the excitation spectra of interacting BEC.

## Discussion

In conclusion, we study the high-momentum excitation of ^133^Cs BEC with widely tunable interactions, and test the universality of Feynman-Tan relation in the description of interaction effect on the excitation spectra of interacting BEC. The Feynman-Tan prediction shows good agreement with the experimental data. Because of the large mass of ^133^Cs atoms relative to ^39^K atoms in the previous experiment^24^, the quantitative explanation for the observed maximum frequency shift confirms the significant role of atomic mass in the resonance frequency shift in Eq. (4). Considering the effective range of *qa* < 3 for the Feynman-Tan relation shown in the experiment of ^39^K BEC, we will prepare the low-density BEC to measure the excitation spectra at the stronger interactions^4^ and check the effective application range of Feynman-Tan relation. In addition, the elementary excitation based on the high-order Bragg diffraction may provide more deep insights for the understanding of inelastic scattering in many-body systems.

## Materials and methods

Experimental setup. As described in Ref. ^34^, we prepare a ^133^Cs BEC through the hybrid evaporation in a trap comprised of magnetic field gradient and several optical dipole trap laser beams. The condensate is then produced in a quasi-1D trap formed mainly from one of these dipole laser beams, which is retro-reflected for driving the Bragg diffraction. We use two acousto-optic modulators (AOMs) in the retro-reflected laser beam, and the frequency detuning ∆*ω*_*res*_ between the counter-propagating Bragg laser beams can be precisely controlled by tuning the frequency difference of the rf driving signals for two AOMs. For the two-photon Bragg diffraction, the zero-momentum BEC is coupled to the momentum state *p* = 2*ħk*, and the frequency detuning is finely scanned around the free-particle excitation energy 4*E*_*R*_*/ħ*. The two-photon Bragg spectroscopy is obtained by measuring the dependence of the fraction of diffrated atoms on the normalized frequency detuning by referring the free-particle kinetic energy ∆*ω* = ∆*ω*_*res*_ − 4*E*_*R*_*/ħ*.

For the second- and third-order Bragg diffractions, the zero-momentum BEC is coupled to the momentum states *p* = 4*ħk* and 6*ħk* through the stimulated four- and six-photon precesses, respectively. In the free-particle excitation, the frequency detunings between the every two counter-propagating photons are 8*E*_*R*_*/ħ* and 12*E*_*R*_*/ħ* in the second- and third-order Bragg spectra, respectively. Due to the distinguishable energy difference, we can implement the second- and third-order Bragg spectra by scanning the frequency detuning around the corresponding free-particle energies. In the experiment, the Bragg coupling is often tuned for guaranteeing that the maximal diffraction fraction is about 10% after 1 ms Bragg pulse.

## Data Availability

All experimental data and any related experimental background information not mentioned in the text are available from the authors upon reasonable request.
